# The care types choice in filial culture: A cross-sectional study of disabled elderly in China

**DOI:** 10.3389/fpubh.2022.954035

**Published:** 2022-09-06

**Authors:** Zheng Zang

**Affiliations:** ^1^School of Marxism, Soochow University, Suzhou, China; ^2^Soochow University Base, Jiangsu Research Centre for Socialist Theory System With Chinese Characteristics, Suzhou, China

**Keywords:** quality of care, older people with disability, informal care, formal care, aging, long-term care, influencing factor

## Abstract

For the past few decades, studies of care types choice have been restricted to the scope of individual characteristics and health status. Meanwhile, the historiography of the research largely ignores the role of filial culture within China. This study sets out to examine the influence of the factors in the cultural context of filial piety on the choice of care types for older people with disability in China. According to the characteristics of filial culture, the factors influencing the choice of care type for the older people in China are summarized as family endowment and support. The study concludes that gender, residence, living alone or not, family income, real estate, pension and community service have momentous effects on the choice of care type of older people with disability; informal care has a substitutive effect on formal care. The research was based on cross-sectional data of CLHLS 2018 and utilized binary logistic regression analysis to compare the factors influencing the choice of old disabled people between formal and informal care. The study implies that in the context of filial culture, the older people's choice of care types is affected by family endowment and community service supply for the older people in China. In the background of filial culture, the government should give informal care official support such as cash and services, so as to change its attribute of private domain of it and enhance the quality of long-term care.

## Introduction

In the study of quality of care, the choice of care types, as an important factor in long-term care, has attracted an increasing attention. There is increasing evidence which suggests that formal care has a higher quality of care than informal one (1–6). This is not only reflected in the assessment of physical and functional health of the care recipient, but also means that the psychological stress of them is reduced due to the reduced mental and life burden of their family members (7–10). Regrettably, in the past, the research on choice of care types in long-term care in Chinese academia focused more on individual characteristics and health status (11–13). And the historiography of the research largely ignores the role of filial culture within China (14, 15).

### Background and problem statement

Under the dual background of aging and filial culture, balancing formal and informal care is one of the effective ways to optimize the long-term care system and meet the caring needs of older people with disabilities (16–18). With the extension of life expectancy and the decline of human fecundity, the proportion of the aging population in China is growing rapidly, which makes China face the challenge of aging population (19). The extension of life expectancy is often accompanied by the decline of self-care ability. In other words, aging and disability occur at the same time (20). Another challenge of the aging population is the huge economic burden of long-term care on families and society (21). Care is one of the core concepts of social policy (22). The World Health Organization (WHO) proposes that long-term care is a system of activities carried out by informal caregivers (family, friends and/or neighbors) and/or professionals (health, social and others), so as to ensure that people who lack full self-care ability can maintain the highest possible quality of life according to their personal priorities and enjoy the greatest possible independence, autonomy, participation, personal enrichment and human dignity (23). In this article, formal care includes social services and nanny care (24, 25). Informal caregivers include spouse, children, grandchildren, daughter-in-law, son-in-law, other relatives, friends and neighbors (26, 27). There is also a care type which is a combination of formal and informal care (28). However, many researchers believe that this is not the mainstream type of care, so it will not be discussed in this article (29, 30).

Current research on formal and informal care is mainly focused on the research in Europe, America, Japan and Korea (6, 17, 25, 26). Fewer researchers in developing countries conducted research on this topic due to young demographic structure and the low pressure of aging. In the last decade or so, due to the rapid development of the aging population in China, Chinese researchers have been conducting research on formal and informal caregiving, drawing on research findings from developed countries (12, 18, 20). There has been much academic debate about the relationship between formal and informal care. Some studies from developed countries have suggested there might be three different relationships between them, which are complementary, alternative and parallel (31–34). According to the view of complementary one, there is a supplementary relationship between formal and informal care, that is, the more family care the older people with disability receive, the more social support they will receive (31). From the perspective of alternative relationship, the increase of informal care will produce a crowding out effect on formal care, which not only reduces the probability of older people with disability entering the nursing home or delays entry (32), but also reduces the use timing and probability of formal care services (33). In the view of parallel relationship, informal and formal care have an impact on the choice of care mode for older people with disability at the same time, rather than mutual influence. After the disabled use formal care, the demand for informal care will decline, but still maintain at a fixed level (34).

### Theoretical framework

Traditionally, China has been dominated by informal care in the form of family care. Formal long-term care in China started late, but there has been a long practice of 'quasi' long-term care services based on institutionalized care of older people (20). In contrast to institutional long-term care, formal home-based and community-based long-term care in China has started to develop in the last decade or so (18). With regard to the long-term care insurance system that accompanies long-term care services, there has been a marked acceleration in the pace of government-led long-term care insurance in China's mainland over the last 5 years (21). At present, the formal care service system for older people with disability in China has not been established, and the existing care service supply cannot effectively meet the caring needs of older people with disability, resulting in the limited substitution of formal care for informal one (13, 15). At the same time, the service contents of formal and informal care are quite different, so it is difficult to realize the perfect replacement of informal care (18). With the influence of traditional culture, especially filial piety culture, informal care is still the main way of care in China (14, 20). However, with the empty nest and fewer children, the supply of informal care represented by family care cannot meet the caring needs of the existing older people with disabilities (21). Some researchers believe that informal care for the older people, as an important part of long-term care, is not free, and may exceed the economic expenditure of formal one (35, 36). Therefore, more and more researchers realize that the evaluation of informal care can help to formulate long-term care policies for its sustainable development (37). At present, the related research mainly includes: the cost of disease care of older people (38), caregiver's economic burden and opportunity cost (39), caregiver's emotional and health cost (40, 41), the trend of informal care cost (42, 43), assessment tools of older people (44), care management (45), the impact of informal care on the health of the older people (46), the comprehensive assessment of the older people (47), the impact of informal care on the health of the older people (43), etc. Informal care faces heavy burden and opportunity cost. More importantly, with the change of traditional concepts and the full opening of the pension service market, formal care services have developed rapidly and become another choice for older people with disability. This article uses CLHLS 2018 (The Chinese Longitudinal Healthy Longevity Survey in 2018) data to analyze the choice of care mode and its influencing factors for the older people with disability in China. Previous studies using CLHLS on older people's care type choices have focused on factors such as gender, education, income, widowhood, ethnic minority status, health status, number of children, and the availability of health insurance and pensions (48–51). However, the information and data collected in the CLHLS on the number of sons, housing status, community services, neighborhood relations and other information of older people in relation to the cultural context of filial piety is often overlooked. Traditional filial culture believes that filial piety is a naturally occurring affection (52). The essence of filial piety, in Mencius' view, is to provide for one's old age (53). As a son or daughter, he or she is obligated to take care of his or her parents, which includes not only taking care of their daily lives, but also comforting their hearts. Whether the children were paying respect and whether they were providing care in times of illness or elderly were most important in determining a sense of filial discrepancy in the parent (54). This feature is reflected in the proverb “Raise children to prevent aging, accumulate grain to prevent hunger.” The culture of filial piety has had a great influence not only in traditional Chinese society, but also in East Asian countries such as Japan and Korea. In traditional East Asian societies, the problem of aging is basically solved within the family (55). In a previous interview with the head of the Japan Welfare Council, when it came to what the biggest challenge of developing a formal long-term care model in Japan was, it was believed that Japan had been traditionally influenced by the Confucian filial culture for a long time making the concept of family-based informal care prevalent. In the midst of rapid aging in Japan, to shift the focus of long-term care from family to society, the need to transform and break through the concept of filial culture was the most difficult and long-term task at that time. In light of this, the article argues that it may be more useful and beneficial in Chinese society to consider cultural factors of filial piety as an influencing factor in the choice of type of care for the older people. The number of activities of daily living (ADL) that cannot be completed is used to measure the disability degree of the older people, and emphasize the functional orientation between informal and formal care among groups with different disability degrees, so as to provide reference for promoting healthy aging.

In this article, we investigate the significant influence of each independent variable on the dependent variable, and test the research hypothesis. The selection of independent variables, in addition to the usual choice of individual characteristics and health status, embodies the characteristics of Chinese filial piety culture mainly in two aspects, namely “raising children for aging” and “living and working in peace and contentment”. “Raising male children for aging” can be reflected in the family endowment represented by the number of sons, family income level, whether to own housing. “Living and working in peace and contentment” can be reflected in whether the community can provide services for the older people. Based on the ideas described above, the following hypotheses were proposed:

**Hypothesis 1 (H1)**. *Different care types have different care effects*.

**Hypothesis 2 (H2)**. *Disabled individuals with different individual characteristics have different preferences for different care types*.

**Hypothesis 3 (H3)**. *Older people with disability with different physical and mental health status have different preferences for care types*.

**Hypothesis 4 (H4)**. *Older people with disability with different family endowments have different preferences for care types*.

**Hypothesis 5 (H5)**. *Whether the community provides services for the older people has an impact on the choice of care types for older people with disability*.

## Materials and methods

The research was based on cross-sectional data of CLHLS 2018 to compare the influencing factors of older people with disability between formal care and informal care.

### Data sources

The data used in this article is from Chinese Longitudinal Healthy Longevity Survey (CLHLS) of Peking University Center for aging health and family research in 2018. The data survey is a follow-up survey of the older people organized by the Research Center for Healthy Aging and Development of Peking University and the National Development Research Institute of China, covering 23 provinces and autonomous regions in China. The respondents are the older people aged 65 and above and the adult family members aged 35–64. The questionnaire is divided into two types: the surviving respondents' questionnaire and the family members of the deceased older people questionnaire. The survey contents of the surviving respondents' questionnaire include the basic situation of the older people and their families, socio-economic background and family structure, economic source and status, self-evaluation of health quality of life, cognitive function, personality and psychological characteristics, daily activities, life-style, life care, disease treatment and medical expenses. The survey contents of the family members of the deceased older people include the time and the cause of death in addition to all the survey content of the surviving one. After the baseline survey in 1998, the survey was conducted in 2000, 2002, 2005, 2008–2009, 2011–2012, 2014 and 2017–2018. The latest follow–up survey data (2017–2018) used in this article interviewed 15,874 older people aged 65 and above, and collected the information of 2,226 older people who died during 2014–2018. CLHLS included a large number of disabled and elderly population samples, and the disability degree of the older people was measured by the Activities of Daily Living Scale (ADLs) and the Instrumental Activities of Daily Living Scale (IADLs), which is helpful to compare the disability degree of the older people in addition. At the same time, CLHLS data is highly representative and reliable.

In this study, the older people who need long-term care were selected according to the six indicators of ADL (bathing, dressing, eating, going to the toilet, controlling defecation and walking indoors) and the time needed to be cared for by others. According to international practice, an older person, who is partially or totally unable to care for himself/herself on at least one of the six indicators or who requires the care of another person for more than 90 days, is considered to be in need of long-term care (7, 9, 12). Through screening from CLHLS 2018, 3510 eligible people were selected as the research sample.

### Variable selection

#### Dependent variable

There are two dependent variables: one is the choice of daily care for the older people with caring needs, including formal and informal care. Formal care includes social services and nanny care (24, 25). Informal caregivers include spouse, children, grandchildren, daughter-in-law, son-in-law, other relatives, friends and neighbors (26, 27). In the initial processing of data, formal care was assigned to 1, while informal care was assigned to 0. The second is the effect of care, including fully meeting the needs of care and not fully meeting the needs of care. The question is measured as the questionnaire “whether the help you get in the six daily activities of e1–e6 can meet your needs” (where e1–e6 stands for the six indicators of ADL) to measure, as the evaluation of control nursing effect. Through the “data conversion” processing, the answer is “fully satisfied” is assigned to 1; the answers are “basically satisfied” and “not satisfied” as “not fully satisfied,” are assigned to 0.

#### Independent variable

According to previous studies (48, 50, 51), and considering the availability of specific data, this article selects a total of 20 independent variables, including individual characteristics, physical and mental health status, family endowment, community services for the older people. The main variables and their assignments are shown in Table 1. The reliability and validity of the collected data were tested using the Cronbach Alpha coefficient and the KMO and Bartlett tests. The reliability of the variables was analyzed using SPSS. The reliability of the variables was 0.829, which was reliable and passed the reliability test, while the KMO coefficient was 0.708, which had good validity and allowed for factor analysis.

**Table 1:** Description of variables.

**Variable category**	**Variable**	**Variable value**	**Mean**	**Standard deviation**
**Dependent variable**	Types of care	Formal care = 1; informal care = 0	0.1452	
	Caring effect	Fully satisfied = 1; not fully satisfied = 0	0.4867	
**Independent variable**	**Individual characteristics**			
	Validated age	Year (continuous numerical variable)	95.24	8.411
	Gender	Male = 1; female = 0	0.3245	
	Years of schooling	Year (continuous numerical variable)	2.98	9.548
	Cohabitation with spouse	Living with spouse = 1; living without spouse = 0	0.1480	
	Current residence	City and town = 1; Rural = 0	0.3028	
	Do you live alone	Non living alone (living with family members or pension institutions) = 1; living alone = 0	0.8347	
	Medical expenses	Non family expenditure = 1; self or family expenditure = 0	0.5235	
	Care expenses	Non family expenditure = 1; self or family expenditure = 0	0.0550	
	**Physical and mental health**			
	Physical health	Level 1–5 (not healthy, not very healthy, middle, relatively healthy, very healthy)	2.3789	1.63555
	Trust in others	Level 1–5 (very distrusting, not very trusting, middle, relatively trusting, very trusting)	3.8621	1.18447
	**Family endowment**			
	Number of male children ever born	Number (continuous numerical variable)	2.80	6.993
	Income level	Level 1–5 (very poor, relatively poor, middle, relatively rich, very rich)	3.0555	0.68208
	Do you own a house	Yes = 1; no = 0	0.8362	
	Do you enjoy retirement benefits	Yes = 1; no = 0	0.2691	
	**Community services for the older people**			
	Daily care services	There are =1; there is no =0.	0.1379	
	On–site medical treatment and medicine delivery service	There are =1; there is no =0.	0.3676	
	Spiritual consolation service	There are =1; there is no =0.	0.1757	
	Daily shopping service	There are =1; there is no =0.	0.1159	
	Organizing social and recreational activities	There are =1; there is no =0.	0.2259	
	Legal aid services	There are =1; there is no =0.	0.2147	
	Provide health knowledge	There are =1; there is no =0.	0.4283	
	Handling neighborhood disputes	There are =1; there is no =0.	0.3115	

### Characteristics of the sample

At present, according to the results of China's seventh census in 2020, the average age of the Chinese population is 38.8 years old (56). Of the Chinese population, 51.24% are male; 48.76% are female (57). In China, 18.70% of the population is aged 60 and over, of which 13.50% is aged 65 and over (58).

Through the analysis of the data, the sample number of older people with disability in CLHLS data in 2018 is 3,510, and the estimated overall disability rate is 22.11%. From the internal structure of the older people with disability, the average age is 95.24 years old. The proportion of the older people is relatively large. What's more, the older they are, the more disabled they are. The proportion of older people with disability in Chinese women is 35.1% higher than that in men, which is 67.55%. The proportion of older people with disability in rural areas is 69.72%, which is 39.44% higher than that in cities and towns. Informal care provided by family members and neighbors is the main care mode for the older people with disability in China, accounting for 85.48%. The proportion of formal social long-term care is relatively small, only 14.52%. In China, 86.20% of older women with disability choose informal care. In rural areas, 93.38% of the older people with disability choose informal care. There are significant gender and urban-rural differences in the long-term care choices of older people with disability in China.

From the perspective of individual characteristics through the analysis of the sample, the average length of education of the older people with disability in China is about 3 years, which is basically equal to the level of primary school. The older people with disability living with their spouses accounts for 30.28%, and the older people with disability living alone accounts for 16.53%. 52.35% of disabled old people enjoy medical insurance, but 94.50% of disabled old people have to pay for care by themselves or their families. From the evaluation of their health status, the vast majority of disabled old people's health status is poor. Because formal care is largely based on trust in others, this article takes trust in others as an independent variable and finds that most older people with disability have higher trust in others.

From the perspective of the background characteristics of filial piety culture through the analysis of the sample, “raising male children for old age” and “living and working in peace and contentment” are two important contents of filial piety culture. In terms of family endowment, the older people with disability in China have an average of 2.8 sons. In addition, 83.62% of the disabled old people own their houses. However, only 26.91% of the older people enjoy retirement insurance benefits. Most old people think their family income is average level. In terms of community services for the older people, only 13.79% of the communities provide daily care services, 17.57% provide spiritual comfort services, and 11.59% provide daily shopping services. 22.59% of the communities will organize community and recreational activities; 21.47% of the communities provide legal aid services. Generally, the three services for the older people provided by the community were health knowledge publicity (42.83%), visiting doctors and drug delivery (36.76%), and mediation of neighborhood disputes (31.15%).

#### Model construction

In this article, when describing the effect of care style for older people with disability, we use the interactive analysis method for the Chi-square test. Because the choice of daily care for older people with disability is a dependent variable, which belongs to a binary variable, the factors influencing the choice of care mode: individual characteristics, health status, family endowment, community service supply for the older people, as control variables, are included in the model for analysis. Therefore, the Binary Logistic Model was used for regression analysis. The Binary Logistic Regression Model was constructed as follows:


(1) $$\begin{array}{r} logit\left(P\right)=ln\frac{P}{1-P}=a+\overset{n}{\underset{i=1}{\sum }}\;\beta_{i}X_{i} \end{array}$$

In this model: P is the probability that the daily care of the older people is formal care. 1–P is the probability of informal care. X_i_ denotes the *i*_th_ influencing factor. β_*i*_ is the partial regression coefficient of the *i*th influencing factor. α is a constant term.

### Results

#### Influencing factors of care satisfaction of older people with disability

According to Table 2, and the Chi-square test, there is no significant difference in formal and informal care satisfaction. Informal care has an alternative effect on formal care. Meanwhile, there are likely significant differences in the satisfaction degree of the older people with different individual characteristics. Among them, age, years of education, living in urban or rural areas, medical insurance, physical health and trust in others all significantly affect the degree of care satisfaction. In terms of family endowments, the number of sons and whether or not to own housing has no significant effect on care satisfaction. However, family income level and whether or not to enjoy retirement benefits significantly likely affect care satisfaction.

**Table 2:** Care effects on older people with disability of different characteristics.

**Variable**	**Proportion of different satisfaction**		**χ^2^**	***P***
	**Fully satisfied (%)**	**Not fully satisfied (%)**		
**Types of care**			0.542	0.461
Formal care	48.59	51.51		
Informal care	50.42	49.58		
**Validated age**			74.168	0.012
**Gender**			2.010	0.156
Male	50.48	49.52		
Female	47.81	52.19		
**Years of schooling**			72.344	0.000
**Cohabitation with spouse**			1.687	0.194
Living with spouse	45.88	54.12		
Living without spouse	49.18	50.82		
**Current residence**			57.719	0.000
City and town	58.64	41.36		
Rural	44.20	55.80		
**Do you live alone**			3.325	0.068
Non living alone (living with family members or pension institutions)	49.44	50.56		
Living alone	45.11	54.89		
**Medical expenses**			21.016	0.000
Non family expenditure	52.53	47.47		
Self or family expenditure	44.23	55.77		
**Care expenses**			0.321	0.571
Non family expenditure	45.45	54.55		
Self or family expenditure	47.80	52.20		
**Physical health**			142.006	0.000
Very healthy	68.60	31.40		
Relatively healthy	61.05	38.95		
Middle	50.22	49.78		
Not very healthy	41.68	58.32		
Not healthy	21.69	78.31		
**Trust in others**			33.648	0.000
Very distrusting	65.18	34.81		
Not very trusting	51.19	48.81		
Middle	41.21	58.79		
Relatively trusting	51.28	48.72		
Very trusting	60.20	39.80		
**Number of male children ever born**			5.830	0.884
**Income level**			199.544	0.000
Very rich	71.11	28.89		
Relatively rich	69.90	30.10		
Middle	47.46	52.54		
Relatively poor	25.62	74.38		
Very poor	25.35	74.65		
**Does it own a house**			0.045	0.832
Yes	48.59	51.41		
No	49.08	50.92		
**Do you enjoy retirement benefits**			39.428	0.000
Yes	58.12	41.88		
No	45.50	54.50		

#### Multiple factors influencing the arrangement of care for the older people

According to the above model, a binary logistic regression analysis using SPSS was conducted to identify the factors influencing the care arrangements for the older people. According to the model fitting information generated by Binary Logistic Regression, χ^2^ = 488.932, significance level (Sig = 0.000) < 0.01, R^2^ = 0.508, which indicates that the model has good fitting degree and good explanatory ability, as shown in Table 3.

**Table 3:** Logistic regression analysis of factors influencing the choice of care types for older people with disability.

**Variable**	**B**	**Sig**.	**Exp (B)**
**Individual characteristics**			
Validated age	0.008	0.543	1.008
Gender	−0.477	0.025	0.620
Years of schooling	0.056	0.009	1.058
Cohabitation with spouse	−0.265	0.423	0.767
Current residence	1.588	0.000	4.892
Do you live alone	−2.089	0.000	0.124
Medical expenses	−0.145	0.490	0.865
Care expenses	0.059	0.896	1.061
**Physical and mental health**			
Physical health	−0.116	0.244	0.890
Trust in others	0.058	0.454	1.060
**Family endowment**			
Number of male children ever born	0.008	0.515	1.008
Income level	0.477	0.002	1.612
Do you own a house	−1.686	0.000	0.185
Do you enjoy retirement benefits	1.018	0.000	2.768
**Community services for the older people**			
Daily care services	0.767	0.006	2.154
On-site medical treatment and medicine delivery service	0.095	0.682	1.100
Spiritual consolation service	0.551	0.048	1.734
Daily shopping service	0.529	0.131	1.698
Organizing social and recreational activities	−0.467	0.156	0.627
Legal aid services	0.320	0.342	1.377
Providing health knowledge	−0.379	0.151	0.685
Handling neighborhood disputes	−1.033	0.002	0.356
**Constant**	−2.422	0.082	0.089
**Chi-square**	488.932		
**-2LL**	743.316		
**Cox and Snell R** ^**2**^	0.306		
**Nagelkerke R** ^**2**^	0.508		

#### The influence of individual characteristics on the choice of care types for older people with disability

From Table 3, we can see that gender, education years, residence and whether living alone have a likely significant impact on the choice of care type of older people with disability. The model showed that there is likely no difference in the choice of care type among age, living with spouse or not, payment method of medical expenses and payment method of care expenses. Female older people with disability tend to choose formal care, which is 1.6 times that of male older people with disability. In addition, the educated older people are more likely to choose formal care, which may be less influenced by traditional filial piety culture such as “raising children to guard against old age.” For the choice of care type, older people with disability living in rural and urban areas are different. The older people with disability living in urban areas are 4.89 times more likely to choose formal care than those living in rural areas. Coupled with the influence of traditional filial piety, most rural older people will mainly focus on informal care. Urban older people are better off and can afford to pay for nannies or aged care facilities, while traditional filial influence is less influential, so they are more likely to choose formal care. Compared with the older people who are not living alone, the older people who live alone are more likely to choose formal care, and the probability is eight times of that of the older people who are not living alone.

#### The influence of family endowment on the choice of care types for older people with disability

It can be seen from Table 3 that family income, whether they own their housing and whether they enjoy retirement benefits have a likely significant impact on their choice of care types. The older people with disability with higher incomes are more likely to choose formal care. The older people with disability without their own housing are more likely to choose formal care, which is 5.4 times of those with real estate. This may be due to the influence of the traditional filial piety culture of “hate to leave a place where one has lived for a long time ” and “living and working in peace and contentment.” The older people with real estate prefer to receive informal care at home. The older people with pension benefits are more likely to receive formal care, which is 2.77 times of those without pension benefits. The number of sons has no significant effect on the choice of care type of older people with disability, indicating that the traditional concept of “raising male children for old age” is likely weakening in China.

#### The impact of community-based services on the choice of care types for older people with disability

It can be seen from Table 3 that “visiting doctors and delivering medicine,” “daily shopping service,” “organizing social and recreational activities,” “providing legal aid activities” and “providing health knowledge” provided by the community have no likely significant impact on the choice of care types for the older people with disability. The “living care service” “spiritual comfort” and “solving neighborhood disputes” provided by the community have a likely significant impact on the choice of care types for older people with disability. If the community provides “living care service” and “spiritual comfort service,” the older people with disability are more likely to choose formal care. In communities providing “neighborhood disputes” services, older people with disability are more likely to choose informal care.

## Discussion and conclusions

Based on the latest 2018 CLHLS data, this article explores the factors that affect the choice of older people with disabilities between formal and informal care. At present, the main ways of caring for the older people in China are informal care based on family and formal care services provided by relying on social resources such as community and pension institutions (20). The study found that: (1) There are differences in the satisfaction degree of the older people with different characteristics. The accessibility of care resources is an important factor affecting the satisfaction of the older people. Family member care is usually the primary choice for the older people. (2) There is no significant correlation between the type of care and the degree of care satisfaction. Informal care has an alternative effect on formal care. (3) Male, rural, non-living alone, low-income, owing real estate, no pension older people tend to choose informal care; female, urban, living alone, high-income, pension, comprehensive community services for the older people tend to choose formal care.

Based on the above conclusion, this article argues that informal care can replace formal one, and that informal care itself is less costly and can reduce the burden on social pensions (59). Therefore, informal care should be encouraged by, giving cash subsidies, proper vacations and social endowment insurance to some caregivers to encourage them to continue to provide informal care (5). These measures could give formal support to informal care, thereby changing the private domain attributes of in-formal care at present. This is a disguised respect for the right to informal care services (60).

Informal care is still the main care model for older people with disability in China nowadays. The results of this study show that 85.48% of older people with disability choose informal care through the analysis of the sample, although the number of male children does not significantly affect the choice of care type for the older people. However, through the analysis of the sample, older people with disability in China now have an average of 2.8 sons, so they still have a broad and realistic basis for playing a role in family care service for older peoples. The choice of informal care for older people with disability may be due to the lack of necessary formal care services (61). However, from the perspective of economy, emotional needs and cultural inheritance, families are still important places for China's older people to provide for aging (18). Although the changes in social and economic development and family structure have weakened the function of family support for thousands of years, social pension services can not completely replace family support, family is the link to maintaining the emotional needs of the older people, and is also the first choice for the older people after their disability (62).

Traditional filial piety culture still has a strong influence in China. Thus, by encouraging the older people to return to the familiar community, with the help of various services and facilities provided by the community, the operation cost of public finance can be reduced and the emotional needs and spiritual dependence of the older people can be satisfied. Influenced by the Chinese culture of filial piety, increasing long-term care services for the older people at the community level and establishing a large number of community-based, fully functional embedded micro-aged care institutions should better meet the needs of the older people at different times and in different self-care situations, allowing them to enjoy continuous professional and personalized services in a familiar environment (63).

Finally, although family care can replace professional care, the long-term care of the older people must depend on the social pension service for older people with disability without children and widowed. In addition, some families will choose formal care because of various reasons (64). Therefore, it is necessary to further improve the long-term care system and to increase the input to formal care institutions. In addition, speeding up the full implementation of the long-term care insurance system can effectively reduce the cost of formal care and enable more older people to have the ability to choose professional care (65).

In the case of China, the traditional culture of filial piety regards long-term care for the older people as one of the main obligations of the family (52). However, as China faces the peak of its aging population, the increase in the number of only-child families since the implementation of family planning policies, the widespread employment of women and the diversification of family living patterns, it may be difficult to sustain a family approach to old age based on the traditional concept of filial piety (20). In this sense, the traditional concept of filial piety is also facing a possible transformation. Long-term care for older people has gradually evolved from a responsibility of traditional family to a practical social issue. What is the current state of demand for long-term care among older people in the context of China's aging population, and how to make the long-term care system work and develop in a healthy way, are questions that the article does not address but that may merit further research. At the same time, although this article analyzes the influencing factors of care types choice in the context of filial culture in China's mainland, it is lacked an analysis about the persistence and challenges to filial piety and care types choice of older people in Hong Kong, which is strongly influenced by the intersection of Chinese and Western cultures (66). In addition, this article is not able to analyze how the cultural meaning and social practice of filial care for aging parents have been transformed in Chinese immigrant families in the Western context (67).

## Data availability statement

The original contributions presented in the study are included in the article/supplementary material, further inquiries can be directed to the corresponding author.

## Author contributions

The author confirms being the sole contributor of this work and has approved it for publication.

## Funding

This work was supported by the Chinese Ministry of Education of Humanities and Social Science Project (Grant Number 19YJCZH235; PI: ZZ), the Borui Special Project of Soochow University on the Creation of a Strong Sense of the Chinese National Community (Grant Number 21BRRT002; PI: ZZ), and the Major Projects of China's Ministry of Education's Key Research Center for Humanities and Social Sciences (Grant Number 17JJD720007).

## Conflict of interest

The author declares that the research was conducted in the absence of any commercial or financial relationships that could be construed as a potential conflict of interest.

## Publisher's note

All claims expressed in this article are solely those of the authors and do not necessarily represent those of their affiliated organizations, or those of the publisher, the editors and the reviewers. Any product that may be evaluated in this article, or claim that may be made by its manufacturer, is not guaranteed or endorsed by the publisher.
